# Myocarditis in a patient treated with Nivolumab and PROSTVAC: a case report

**DOI:** 10.1186/s40425-018-0473-0

**Published:** 2018-12-18

**Authors:** Cecilia Monge, Hoyoung Maeng, Alessandra Brofferio, Andrea B. Apolo, Bharath Sathya, Andrew E. Arai, James L. Gulley, Marijo Bilusic

**Affiliations:** 1 Medical Oncology Service, Center for Cancer Research, National Cancer Institute, National Institutes of Health, Bethesda, MD USA; 2 Vaccine Branch, Center for Cancer Research, National Cancer Institute, National Institutes of Health, Bethesda, MD USA; 3 Cardiovascular Branch, National Heart, Lung, and Blood Institute, National Institutes of Health, Bethesda, MD USA; 4 Genitourinary Malignancies Branch, Center for Cancer Research, National Cancer Institute, National Institutes of Health, 10 Center Dr., Rm. B2L312, Bethesda, MD 20892 USA

## Abstract

**Background:**

Immune checkpoint inhibitors have revolutionized treatment and improved survival in many cancers. However, since immune-related adverse events (irAEs) are potentially fatal, early recognition and prompt treatment are warranted. One of the rarest but most dramatic irAE is myocarditis, which has significant morbidity and mortality if not recognized and treated early.

**Objective:**

To report the first case of myocarditis in a patient with metastatic castration-resistant prostate cancer (mCRPC) treated with a combination of nivolumab, an anti-programmed cell death protein 1 antibody, and PROSTVAC, a vector-based therapeutic prostate cancer vaccine.

**Case Report:**

A 79-year-old man with mCRPC metastatic to bone and lymph nodes and a history of atrial fibrillation presented with blurred vision and pain and stiffness in the upper back after 8 weeks on a clinical trial with nivolumab (1 mg/kg) and PROSTVAC, both given every 2 weeks. Eye exam was within normal limits, while musculoskeletal exam revealed tenderness in trapezius muscles and decreased motor strength in arms (III/V) and neck (IV/V). The rest of the physical exam was within normal limits, with the exception of an irregular heart rhythm. Laboratory tests were as follows: creatinine kinase (CK) 3200 U/L (normal: 39–308 U/L), CK-MB 65.7 mcg/L (normal: 0–7.6 mcg/L), troponin I 0.209 ng/mL (normal: 0–0.056 ng/mL). Electrocardiogram (ECG) revealed atrial fibrillation with QT prolongation (QTc 514 msec) and left anterior fascicular block, unchanged from baseline. 2D-echocardiogram showed a left ventricular ejection fraction of 65% with an enlarged left atrium, dilated right ventricle, and increased pulmonary artery pressure (45 mmHg). ProBNP was elevated at 1463 pg/mL and peaked at 3066 pg/mL one day after hydration. With a presumed diagnosis of autoimmune myositis and possible myocarditis, the patient was admitted and started on methylprednisolone 1 mg/kg/day. Cardiac MRI showed elevated native myocardial T1 values consistent with myocarditis (Fig. 1). The patient was discharged on a prednisone taper after normalization of cardiac enzymes on day 4. Treatment with PROSTVAC continued for three more months; nivolumab was discontinued. Six months later, patient is doing well, with no residual cardiac damage.

**Discussion:**

Cardiovascular irAEs are relatively rare (< 1%) and have a variety of clinical presentations. Myocarditis is potentially life-threatening and can range from subclinical to fulminant. Therefore, clinical suspicion, early detection, and prompt treatment are imperative (1). The initial diagnostic workup should include cardiac enzymes, ECG, and 2D-echocardiogram. The most commonly observed ECG changes are generalized repolarization abnormalities, prolonged QT interval, and conduction abnormalities (2). An elevated troponin I in the absence of overt coronary artery disease is suggestive of myocarditis and should be evaluated further. Myocardial biopsy is the standard diagnostic procedure; however, a cardiac MRI can achieve a diagnosis when biopsy is not feasible (3). Advancements in parametric mapping techniques have allowed the use of native myocardial T1 in the detection of myocarditis, as it has superior diagnostic performance and higher sensitivity than older parameters (3). Our patient had been treated with an immune checkpoint inhibitor and a therapeutic cancer vaccine to induce effective antitumor activity through immunogenic intensification and presented with muscle stiffness and elevated CK. Although he had no new cardiovascular symptoms, cardiac enzymes were tested to rule out myocardial involvement. MRI with gadolinium confirmed the diagnosis of myocarditis. To date, none of the 1360 patients treated with PROSTVAC as a single agent have developed myocarditis, while myocarditis has been rarely reported in patients treated with nivolumab (< 1%) (1). Whether the combination of PROSTVAC and nivolumab presents an additional risk of myocarditis is unclear. To our knowledge, this is the first case of myocarditis in a patient with mCRPC receiving simultaneous treatment with an immune checkpoint inhibitor and a prostate cancer vaccine.

Our experience highlights the importance of suspicion and early intervention in patients who present with cardiac abnormalities after receiving cancer immunotherapy. We propose following protocol: baseline troponin, ECG, and 2D-echocardiogram prior to treatment, then repeated troponin at 2, 4, and 12 weeks post-treatment, then monthly. If troponin becomes positive without alternative explanation, myocarditis should be ruled out with cardiac MRI or myocardial biopsy, and patient should be admitted for treatment with high-dose steroids as early intervention may minimize myocardial injury.

## Background

Immune checkpoint inhibitors have revolutionized treatment and improved survival in many cancers. However, since immune-related adverse events (irAEs) are potentially fatal, early recognition and prompt treatment are warranted. Immune myocarditis exemplifies the significance of such a need highlighting the importance of raising the awareness of the risk and condition that will lead to an expedited intervention.

## Objective

To report the first case of myocarditis in a patient with metastatic castration-resistant prostate cancer (mCRPC) treated with a combination of nivolumab, an anti-programmed cell death protein 1 antibody, and PROSTVAC, a vector-based therapeutic prostate cancer vaccine targeting prostate specific antigen (PSA).

## Case report

A 79-year-old man with mCRPC metastatic to bone and lymph nodes and a history of atrial fibrillation presented with blurred vision and pain and stiffness in the upper back after 8 weeks on a clinical trial with nivolumab (1 mg/kg) and PROSTVAC, both given every 2 weeks. Eye exam was within normal limits, while musculoskeletal exam revealed tenderness in trapezius muscles and decreased motor strength in arms (III/V) and neck (I*V*/V). The rest of the physical exam was within normal limits, with the exception of an irregular heart rhythm. Laboratory tests were as follows: creatinine kinase (CK) 3200 U/L (normal: 39–308 U/L), CK-MB 65.7 mcg/L (normal: 0–7.6 mcg/L), troponin I 0.209 ng/mL (normal: 0–0.056 ng/mL). Electrocardiogram (ECG) revealed atrial fibrillation with QT prolongation (QTc 514 msec) and left anterior fascicular block, all unchanged from baseline three months before. 2D-echocardiogram showed a left ventricular ejection fraction of 65% with an enlarged left atrium, a dilated right ventricle and increased pulmonary artery pressure (45 mmHg). ProBNP was elevated at 1463 pg/mL and peaked at 3066 pg/mL one day later after hydration. With a presumed diagnosis of autoimmune myositis and possible myocarditis, the patient was admitted and started on methylprednisolone 1 mg/kg/day.

**Fig. 1:**
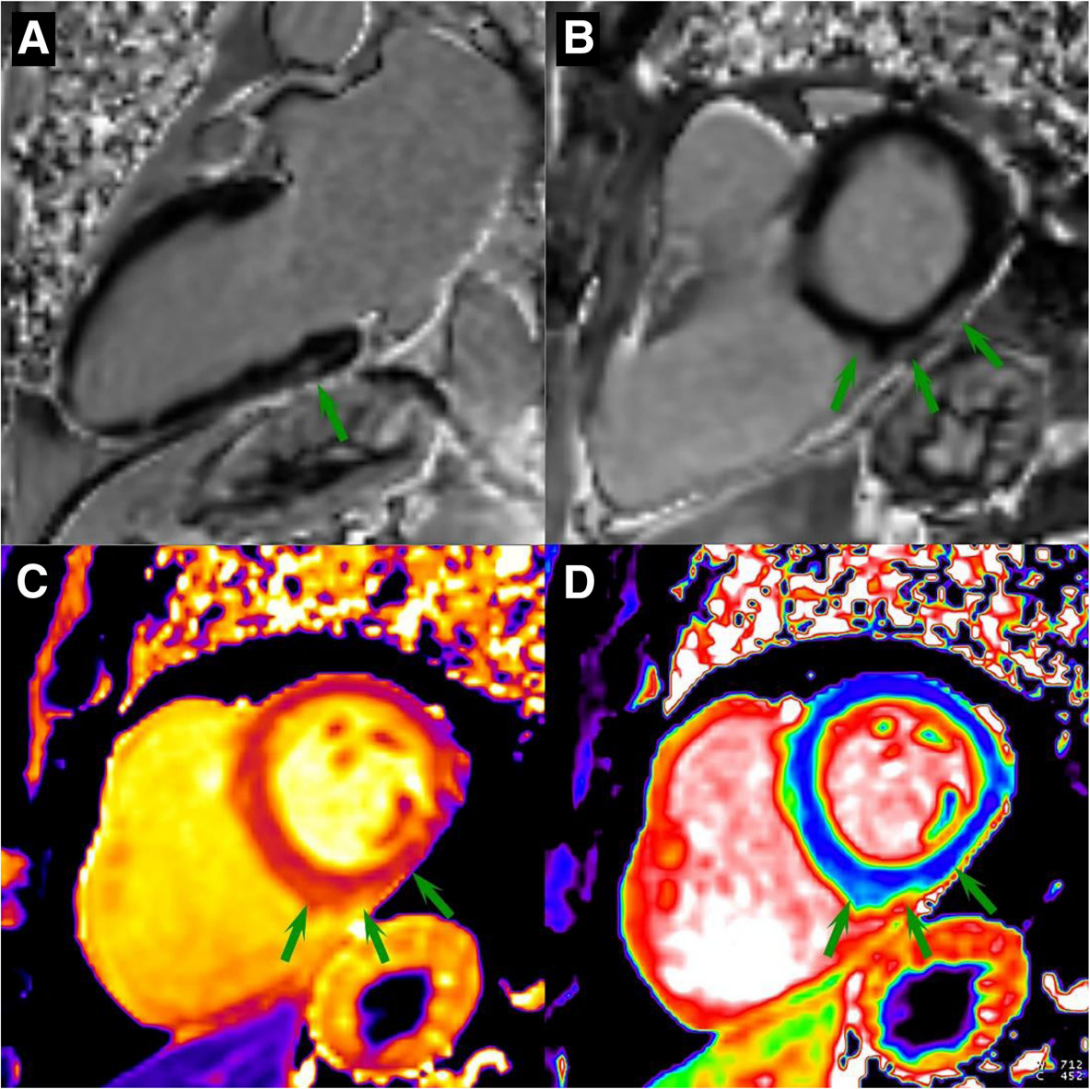
CMR findings support a diagnosis of myocarditis. Late gadolinium enhancement (LGE) images in long axis (**a**) and short axis (**b**) views show epicardial enhancement in the basal inferior wall and inferior septum (arrows). Native T1 (**c**) and extracellular volume fraction (**d**) were abnormal in the basal inferior wall and inferior septum. Mid inferior myocardial T2 was at the borderline between normal and mildly abnormal (not show). Early gadolinium enhancement (not shown) was also abnormal in a similar but more extensive distribution than the LGE

The cardiac MRI findings were consistent with myocarditis involving small areas of the myocardium. Left ventricular size and function were normal (ejection fraction 59%) with normal regional wall motion. Patches of late gadolinium enhancement (LGE) were seen in the basal and mid inferior wall showing an epicardial pattern compatible with myocarditis. Early gadolinium enhancement was abnormal in a similar distribution to the LGE but more extensive. Myocardial T1 was 1411 ms. in the basal inferoseptum and 1231 ms. in the mid inferior wall (normal native T1 < 1350 ms.). Myocardial T2 was normal in all segments except the mid inferior wall where it was at the borderline between normal and mildly elevated. Extra cellular volume fraction (ECV) was more diffusely abnormal in the basal inferior wall 41.1% (normal 25.5, 95% confidence intervals 20.5–30.5%) in the closest region of interest to the area of abnormal LGE but also diffusely mildly elevated (32–33%) in several basal and mid ventricular segments (Fig. 1).

The patient was discharged on a multiple week oral prednisone taper after normalization of cardiac enzymes on day 4. Treatment with PROSTVAC continued for three more months; nivolumab was discontinued. Six months later, patient is doing well, with no residual cardiac damage.

## Discussion

Cardiovascular irAEs are uncommon (approximately 1%) and have a variety of clinical presentations. Myocarditis is potentially life-threatening and can range from subclinical to fulminant. Therefore, clinical suspicion, early detection, and prompt treatment are imperative [1–3]. The initial diagnostic workup should include cardiac enzymes, ECG, and 2D-echocardiogram. The most commonly observed ECG changes are generalized repolarization abnormalities, prolonged QT interval, and conduction abnormalities [4]. An elevated troponin I in the absence of overt coronary artery disease is suggestive of myocarditis and should be evaluated further. Myocardial biopsy is the standard diagnostic procedure; however, a cardiac MRI can achieve a diagnosis when biopsy is not feasible. Cardiac MRI imaging in conjunction with the Lake Louis Criteria have a sensitivity of 74% and a specificity of 86% [5]. Our patient had been treated with an immune checkpoint inhibitor and a therapeutic cancer vaccine to induce effective antitumor activity through immunogenic intensification and presented with muscle stiffness and elevated CK. Although he had no new cardiovascular symptoms, cardiac enzymes were tested to rule out myocardial involvement. MRI with gadolinium confirmed the diagnosis of myocarditis. To date, none of the 1360 patients treated with PROSTVAC as a single agent have developed myocarditis, while myocarditis has been reported in patients treated with nivolumab (approximately 1%) [1, 3]. Whether the combination of PROSTVAC and nivolumab presents an additional risk of myocarditis is unclear. To our knowledge, this is the first case of myocarditis in a patient with mCRPC receiving simultaneous treatment with an immune checkpoint inhibitor and a prostate cancer vaccine.

Our experience highlights the importance of suspicion, diagnosis and early intervention in patients who present with cardiac abnormalities and possible myocarditis after receiving immunotherapy [6]. Prompt treatment with high-dose steroids may minimize myocardial injury.
